# Evaluating the Different Stages of Parkinson’s Disease Using Electroencephalography With Holo-Hilbert Spectral Analysis

**DOI:** 10.3389/fnagi.2022.832637

**Published:** 2022-05-10

**Authors:** Kuo-Hsuan Chang, Isobel Timothea French, Wei-Kuang Liang, Yen-Shi Lo, Yi-Ru Wang, Mei-Ling Cheng, Norden E. Huang, Hsiu-Chuan Wu, Siew-Na Lim, Chiung-Mei Chen, Chi-Hung Juan

**Affiliations:** ^1^Department of Neurology, Chang Gung Memorial Hospital, Chang Gung University College of Medicine, Taoyuan, Taiwan; ^2^Institute of Cognitive Neuroscience, National Central University, Taoyuan, Taiwan; ^3^Taiwan International Graduate Program in Interdisciplinary Neuroscience, National Central University and Academia Sinica, Taipei, Taiwan; ^4^Cognitive Intelligence and Precision Healthcare Research Center, National Central University, Taoyuan, Taiwan; ^5^Department of Biomedical Sciences, Chang Gung University, Taoyuan, Taiwan; ^6^Metabolomics Core Laboratory, Healthy Aging Research Center, Chang Gung University, Taoyuan, Taiwan; ^7^Clinical Phenome Center, Chang Gung Memorial Hospital, Taoyuan, Taiwan; ^8^Data Analysis and Application Laboratory, The First Institute of Oceanography, Qingdao, China

**Keywords:** electroencephalography, Holo-Hilbert spectral analysis, machine learning, Parkinson’s disease, depression

## Abstract

Electroencephalography (EEG) can reveal the abnormalities of dopaminergic subcortico-cortical circuits in patients with Parkinson’s disease (PD). However, conventional time-frequency analysis of EEG signals cannot fully reveal the non-linear processes of neural activities and interactions. A novel Holo-Hilbert Spectral Analysis (HHSA) was applied to reveal non-linear features of resting state EEG in 99 PD patients and 59 healthy controls (HCs). PD patients demonstrated a reduction of β bands in frontal and central regions, and reduction of γ bands in central, parietal, and temporal regions. Compared with early-stage PD patients, late-stage PD patients demonstrated reduction of β bands in the posterior central region, and increased θ and δ2 bands in the left parietal region. θ and β bands in all brain regions were positively correlated with Hamilton depression rating scale scores. Machine learning algorithms using three prioritized HHSA features demonstrated “Bag” with the best accuracy of 0.90, followed by “LogitBoost” with an accuracy of 0.89. Our findings strengthen the application of HHSA to reveal high-dimensional frequency features in EEG signals of PD patients. The EEG characteristics extracted by HHSA are important markers for the identification of depression severity and diagnosis of PD.

## Introduction

Parkinson’s disease (PD) is a neurodegenerative disease affecting the brain, predominantly pigmented nuclei in the midbrain, brainstem, cerebral cortex and olfactory tubercle (Braak et al., 2003). Other than motor symptoms, PD also presents cognitive symptoms, which usually occur during more advanced stages of disease or may coincide with motor symptoms if there is a disruption of fronto-striatal circuits (Marsden, 1982; Cooper et al., 1991). Although the neurodegeneration of PD occurs mainly in subcortical structures, dopaminergic cortical-subcortical circuits between the basal ganglion, thalamus, and frontal lobes are also affected (Alexander et al., 1986). The disruption of these circuits leads to specific cognitive deficits in patients with PD.

The activity of cortical neurons averaged over the cortex can be illustrated using electroencephalogram (EEG) (Nunez et al., 2001). EEG signals can be considered as brain-computer interface systems (BCI), as EEG-based intelligent BCI enables the uninterrupted monitoring of fluctuations in human cognitive states and is beneficial for healthcare support and research in various fields. This system registers the capability of human brain interaction with the environment and advanced technology via machine learning algorithms. EEG signals directly measure cortical electrical activity with high temporal resolution (Ramadan and Vasilakos, 2016) and is the most largely used non-invasive modality for both real-world BCIs and clinical use (Schalk et al., 2004). With comparatively high signal quality, reliability and mobility compared to other imaging approaches, EEG devices collect signals in non-overlapping frequency bands, where their powerful intra-band connection reflects distinct behavioral states (Zhang et al., 2017), and present diverse corresponding features and motifs. Moreover, the temporal resolution is exceedingly high, up to the millisecond level, with minimal risk compared to other invasive and non-invasive modalities. Nonetheless, a drawback of the EEG is the low spatial resolution within signals ensuing from the limited number of electrodes. Hence, this obligates consideration of the inferior signal-to-noise ratio since objective factors like environmental noise and subjective factors like fatigue status could contaminate the EEG signals. Thus, a broad category of unsupervised learning algorithms for signal enhancement, namely blind source separation, estimates original sources and parameters of a mixing system and removes artifact signals, including eye blinks and movement (Sweeney et al., 2012). Independent component analysis (ICA) is the most widely used blind source separation (BSS) method as it decomposes observed signals into independent components and restructures clean signals by eradicating independent components comprising artifacts (Gu et al., 2021). Machine learning has been incorporated into EEG signals’ analysis, and is a subset of computational intelligence comprising numerous research areas. Machine learning depends on general patterns of reasoning via computer systems to investigate a specific task without providing obvious coded instructions. In supervised learning, it divides the data into two subsets during the learning process: a training set (i.e., dataset to train a model) and a test set (i.e., dataset to test the trained model). Supervised learning can be used for classification and regression by applying what has been learned in the training stage using labeled examples to test the new data (i.e., testing data) to classify types of or predict future events. Contrariwise, unsupervised learning is utilized when the data used for training are neither classified nor labeled (Kasabov, 2001). In EEG-based BCI applications, numerous model types have been used and developed for machine learning, where prominent families of models comprise linear classifiers, neural networks, non-linear Bayesian classifiers, nearest neighbor classifiers, and classifier combinations (Kotsiantis et al., 2006). To apply machine learning algorithms to EEG data, EEG signals must be pre-processed and their features extracted from raw data, including frequency band power and connectivity features between two channels (Daly et al., 2012). The training data used to train the classifier and test data for estimating the classifier belong to the same feature space and follow the same probability distribution (Gu et al., 2021).

Following the above, studies show that EEG is useful in identifying alterations in electrical activity in the brains of PD patients. Han et al. (2013) analyzed EEG signals in patients with PD and healthy controls (HCs), and found increased powers in θ and δ bands, and reduced powers in the α and β bands. Benz et al. (2014) discovered significant differences in EEG activity between patients with PD and Alzheimer’s disease (AD), with more pronounced slowing of EEG in patients with PD compared to AD group (Benz et al., 2014). Babiloni et al. (2011) mapped eye-closed resting state EEG (rsEEG), and found abnormal alterations of δ bands at central regions, as well as θ and β bands at posterior cortical regions. Cao et al. (2021) used event-related spectral perturbation analysis to investigate EEG spectral dynamics induced by different walking phases and distinguished EEG signals throughout the transition from walking to voluntary stopping from those during the transition to involuntary stopping caused by freezing of gait (Cao et al., 2021). However, EEG signals in the above studies were only inspected visually based on a set of qualitative rules with subjective interpretations (Ebersole and Pedley, 2003). The non-linear and non-stationary processes of neural activities and interactions cannot be fully revealed with conventional time-frequency analysis based on linear Fourier and Wavelet transforms (Huang et al., 2016). Development of a new models for EEG signal analysis is thus necessary to detect information about neuronal firings and their interactions, including cross-scale coupling of neural networks through synchronizations, resonance, phase locking, and amplitude modulations (AM). Fuzzy models, which apply fuzzy rules, fuzzy logic, and fuzzy measure theory (i.e., fuzzy integrals) to a fuzzy inference system, are better for processing non-linear and non-stationary EEG signals in BCI research (Gu et al., 2021). As such, this has been widely used in entropy analysis to measure the dynamic complexity of signals, and is a crucial and urgent development as the state of complexity in humans is significantly affected by health. Cao and Lin (2018) demonstrated that inherent fuzzy entropy (Inherent FuzzyEn) and its multiscale version, which utilized empirical mode decomposition (EMD) and fuzzy membership function (i.e., exponential function), addresses the dynamic complexity in EEG data (Cao and Lin, 2018). This method was also applied successfully in investigating the extraction of repetitive steady-state visual evoked potentials to investigate EEG complexity change in patients with migraine (Cao et al., 2020).

Adhering to this, recent studies systematically demonstrate that the Holo-Hilbert spectrum analysis (HHSA) can reveal dimensional and non-linear characteristics of EEG signals in the domain of visual perception (Nguyen et al., 2019; Juan et al., 2021) and working memory (Liang et al., 2021), and outperformed conventional linear analytical methods (i.e., Fourier and Wavelet analyses). HHSA is an innovative investigation instrument based on EMD and Hilbert Huang Transformation (Huang, 1998; Huang et al., 2016) which delivers an informational and high-dimensional frequency illustration of data from non-stationary and non-linear processes. This comprehensive method permits the investigation of the carrier and AM frequencies, as well as their interactions in neuronal oscillations. This approach is particularly important to further elucidate differences in non-linear neural processing of the envelope in AM signals in PD patients and HCs, thus providing potential neurodegenerative signals within the cortex of patients with PD.

Through this analysis, we desire to detect a decrease in higher frequency and increase in lower frequency powers, as indicated in previous reports (Tanaka et al., 2000; Kotini et al., 2005; Bosboom et al., 2006; Moazami-Goudarzi et al., 2008). Differences in the rsEEG of PD patients could also yield an impact on their cognitive or psychiatric status (Soikkeli et al., 1991; Caviness et al., 2007). Hence, in this study, we analyzed eye opening and closing rsEEG in age- and sex-matched patients with PD and HCs using the HHSA as this method can divulge the non-linear and non-stationary processes of neural activities and interactions of the rsEEG in both groups. We then looked for whether there were any association of the rsEEG with clinical assessments in both groups, and correlated the HHSA results with clinical and psychiatric scale scores. The HHSA features extracted from EEG signals were further analyzed by machine learning algorithms to generate a predictive model to distinguish between PD patients and HCs.

## Materials and Methods

### Patient Recruitment

This is a cross-sectional study were patients were recruited during 2018/07/01 to 2020/12/31 in Chang Gung Memorial Hospital-Linkou Medical Center in Taiwan. Patients were diagnosed with PD according to the UK Brain Bank criteria for PD Demographic information, Levodopa Equivalent Daily Dose (LEDD) (Tomlinson et al., 2010), the Unified Parkinson’s Disease Rating Scale (UPDRS) (Goetz, 2003) and Hoehn and Yahr (H&Y) stage (Hoehn and Yahr, 1967) were recorded for each patient. All patients underwent a battery of neuropsychological assessments including the Mini-Mental State Examination (MMSE) (Tombaugh and McIntyre, 1992), Montreal Cognitive Assessment (MoCA) (Nasreddine et al., 2005), Clinical Dementia Rating (CDR) (Morris, 1993), Beck Depression Inventory II (BDI-II) (Beck et al., 1996), Hamilton Depression Rating Scale (HAM-D) (Hamilton, 1986), Activities of Daily Living (ADL) (Lawton and Brody, 1969), the Parkinson’s Disease Questionnaire (PDQ-39) (Jenkinson et al., 1995), and Neuropsychiatric Inventory Questionnaire (NPI) (Cummings, 1997). Patients with PD (H&Y stage 1–2) were defined as those in early stage (EPD), while those with H&Y stage greater than 2 were classified as those in late stage (LPD). Sex- and age-matched HCs were randomly recruited from neurology outpatient clinics. All subjects had no systemic infection, chronic renal failure, cardiac or liver dysfunction, malignancies, autoimmune diseases, stroke, or neurodegenerative diseases other than PD. Diagnoses were determined by two experienced neurologists in movement disorders (K. H. Chang and C. M. Chen) who were blinded to both EEG and neuropsychiatric results.

### Electroencephalography Acquisition Protocol

Electroencephalography data acquisition was performed using the Brain Products GmbH amplifier (Brain Amp) with a 32-channel EEG cap (EASYCAP) according to the international 10–20 system. Both caps were saturated with Ag/AgCl gel and placed on all participants’ heads. The whole 10-min for both eye-closed and eye-opened resting EEG were digitized at a 2,500 Hz (5 PD) and 5,000 Hz (59 HCs, 95 PD) sampling rate without any online filters. The reference was the average of electrodes at the two sides of the mastoid (A1 and A2) or POZ. Two pairs of bipolar electrodes were also mounted to detect eye movements with the VEOU and VEOL electrodes placed above and below the left eye, respectively, with the HEOR and HEOL electrodes positioned adjacent to the canthus of each eye. The impedances of all channels were maintained below 5 kΩ.

### Electroencephalography Recording, Preprocessing, and Denoising

Electroencephalography recordings were all downsampled to 2500 Hz and re-referenced to the frontal cephalic (Fz) channel, and further standardized to 26 channels (FP1, FP2, F7, F3, Fz, F4, F8, FC1, FC2, FC5, FC6, C3, Cz, C4, CP1, CP2, CP5, CP6, P3, P7, Pz, P4, P8, O1, Oz, O2). The data were then fragmented into consecutive epochs of 8000 ms. EEG epochs with ocular, muscular, and other artifacts were preliminarily identified and excluded by a computerized automatic procedure using ICA. The HHSA was then used to compute the power spectrum of each trial.

### Holo-Hilbert Spectral Analysis for Electroencephalography Recordings

**FIGURE 1:**
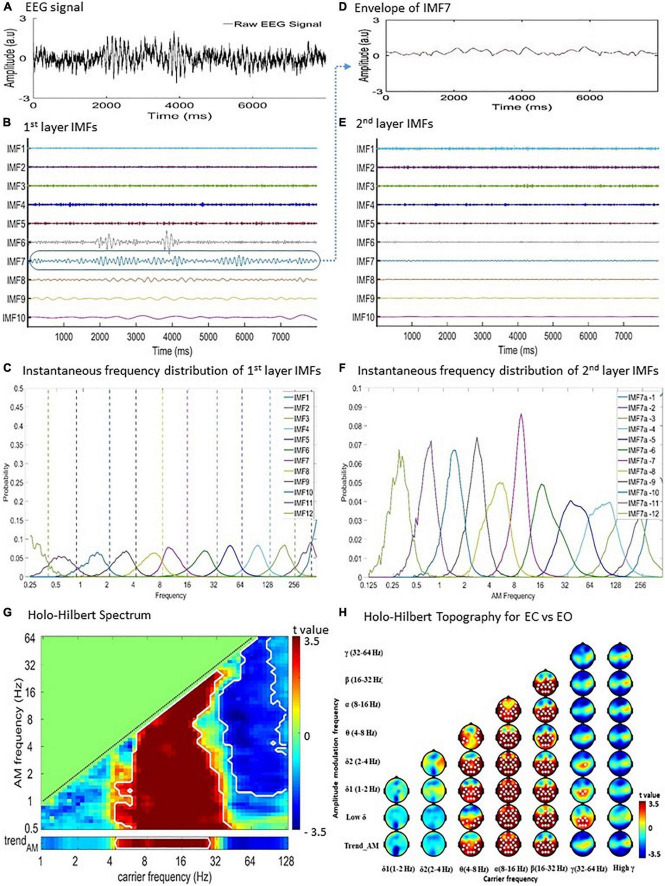
Holo-Hilbert Spectral Analysis (HHSA) for the EEG recordings. Diagram of two-layer ensemble empirical mode decomposition (EEMD) of resting EEG data. **(A)** Raw EEG signal from a single subject at a single channel. **(B)** The first layer EEMD decomposes the raw signal into 12 intrinsic mode functions (IMFs). **(C)** The instantaneous frequency distribution of first layer IMFs denoting the frequency ranges represented by each IMF. **(D)** To illustrate the second layer EEMD, the envelope of IMF7 was extracted. **(E)** Subsequent application of EEMD on the IMF7 envelope produces the second layer IMFs. **(F)** Instantaneous frequency distribution of the second layer IMFs designating the frequency ranges represented by each IMF. **(G)** Holo-Hilbert spectrum of the carrier wave modulated by the envelopes. **(H)** Summated topographical maps of AMs of the carrier wave (0.5–64 Hz) by envelopes (1–128 Hz) frequencies. EC, eye closing; EO, eye opening.

Holo-Hilbert spectral analysis is an analytical method derived from Hilbert-Huang Transform (HHT) for analyzing complex signals such as EEG (Huang et al., 1998, 2016; Nguyen et al., 2019; Liang et al., 2021). HHT was achieved by using EMD and estimating instantaneous frequency by Hilbert transform. The EMD decomposed data into a finite number [∼log_2_ (length of data)] of intrinsic mode functions (IMFs) and generated a high-resolution time-frequency spectral representation (Figures 1A–E).

In EMD, each IMF is obtained by a sifting process with the following properties: (1) the number of local extrema (including local maxima and local minima) and the number of zero-crossings must either be equal or differ by up to 1; and (2) the mean value of the envelope estimated by local maxima and local minima should be zero. Based on EMD, the advanced HHSA is achieved using a process of two-layer EMD of natural signals and high-dimensional spectral representation. In the current study, both the first and second layer EMD were performed using an improved complete ensemble EMD with adaptive noise (CEEMDAN) method for obtaining the first and second layer IMFs (Colominas et al., 2012, 2014; Tsai and Liang, 2021). Compared with the original EMD or ensemble EMD method, the improved CEEMDAN method has characteristics of less mode-mixing, lower reconstruction error (i.e., the noise residual within IMFs) (Wu and Huang, 2009), and higher consistency of frequency distribution ranges in the order of IMFs for different noisy signals (Colominas et al., 2014; Tsai and Liang, 2021). The steps of the two-layer CEEMDAN are described as follows:

Apply the first layer CEEMDAN to data of each EEG channel to decompose the data into a collection of IMFs (Figure 1B). The first layer EMD can be expressed as:


$$x\,\left(t\right)=\sum_{j=1}^{n}c_{j}\,\left(t\right)+r_{n}=\sum_{j=1}^{n}a_{j}\,\left(t\right)\,\cos\theta_{j}\,\left(t\right)+r_{n},$$

in which the signal *x*(*t*) is decomposed into *n* IMFs, and the *j*th IMF, *c*_*j*_(*t*), is further expressed as *a*_*j*_(*t*)*cos*⁡θ_*j*_(*t*) , where *a*_*j*_(*t*) is the amplitude function (AF) achieved using a cubic spline algorithm, θ_*j*_(*t*) is the phase function (PF) obtained using a direct quadrature (DQ) transform (Huang et al., 2009), and *r_n* is the final residue (i.e., trend) without any oscillatory characteristics. The instantaneous frequency (IF) of the *j*th IMF is obtained by taking the time derivative of the phase function, θ_*j*_(*t*).

Perform the second layer CEEMDAN on the AF (i.e., envelope) of each IMF acquired from the first layer EMD (see Figures 1D,E), given as:


$$a_{j}\,\left(t\right)=\sum_{k=1}^{l_{2}}a_{j\,k}\,\left(t\right)\,\cos\Theta_{j\,k}\,\left(t\right)+R_{j\,l_{2}},$$

where the *j*th first layer IMF’s AF, *a*_*j*_(*t*), is decomposed into *l*_2_ second layer IMFs, and each second layer IMF is further expressed as *a*_*jk*_(*t*)*cos*⁡Θ_*jk*_(*t*) , where *a*_*jk*_(*t*) is the second layer AF, Θ_*jk*_(*t*) is the second layer PF, and *R*_*jl_2*_ is the second layer final residue without rhythmic characteristics. Therefore, these second layer IMFs expand each first layer AF in terms of rhythmic AMs from small to large time scales. The nested form of the entire two-layer CEEMDAN are:


$$x\,\left(t\right)=\sum_{j=1}^{n}\left[\sum_{k=1}^{l_{2}}a_{j\,k}\,\left(t\right)\,\cos\Theta_{j\,k}\,\left(t\right)+R_{j\,l_{2}}\right]\,\cos\theta_{j}\,\left(t\right)+r_{n}.$$

By taking the time derivative of the second layer PF, Θ_*jk*_(*t*), we obtained the instantaneous “AM frequency” (Figure 1F). To highlight the concept of instantaneous “AM frequency” derived from the second layer CEEMDAN, the original IF obtained from the first layer CEEMDAN will be referred to as the instantaneous “carrier frequency” when it is represented in a spectrum.

**FIGURE 2:**
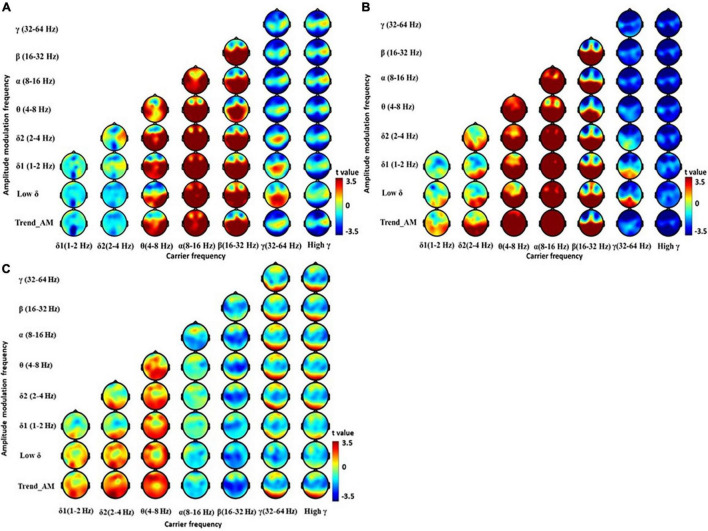
Electroencephalography power difference between PD and HC using Holo-Hilbert Spectrum Analysis (HHSA). Holo-Hilbert topography (HHT) in the eyes-closed minus the eyes-open condition based on cluster-based non-parametric permutations in the **(A)** HC, **(B)** PD, and **(C)** PD minus HC. The color bar denotes *t*-statistics ranging from blue (–3.5) to red (+3.5).

Given that all the oscillatory information was obtained, such as the first and second layers of AF, instantaneous frequency, and instantaneous AM frequency (including instantaneous phase, and instantaneous AM phase), the spectral representation can be achieved as follows:

(A) The AM power (i.e., square of the second layer AF) of each second layer IMF for every specific time point is projected to the spectrum according to the instantaneous AM frequency of the second layer IMF, and the instantaneous frequency of its corresponding first layer IMF, resulting in the 3D HHS. The coordinate of “carrier frequency” is consistent with the frequency coordinate in conventional time-frequency spectrograms.
(B) Take the marginal sum/mean of the 3D HHS (1) over the AMF axis (or a specific range of the AMF axis, Figure 1E); (2) over the time axis (or a specific window of the time axis); or (3) over the carrier frequency axis (or a specific range of the carrier frequency axis, Figure 1C). This will result in the 2D time-carrier frequency, carrier-AM frequency, or time-AM frequency marginal HHS, respectively. This optional step could be tailored to specific research interests. For the current resting EEG study, the marginal sum is taken over the entire time axis to produce the 2D carrier-AM frequency marginal HHS.

In the 2D carrier-AM frequency marginal HHS, AMF should be lower than carrier frequency (i.e., $\frac{d\,\Theta_{j\,k}}{d\,t}<\frac{d\,\theta_{j}}{d\,t}$) because for any given IMF the rhythmic amplitude variations (i.e., AMs) should be slower than its corresponding carrier wave. Therefore, AM power can only exist below the carrier-AM frequency “equi-frequency” line on the HHS (Figure 1G). In the present study both the carrier and AM frequencies are log_2_-scaled. The lowermost AM frequency bin, denoted as “trend_*AM*_,” is positioned at the bottom of the HHS, separated from other higher AM frequency bins. The spectral power in the trend_*AM*_ bin signifies the “unmodulated” power estimated by the trend (i.e., the last component) of each second-layer EMD. Both AM and carrier frequency bins are categorized according to physiological frequencies as following: low δ (0.5–1 Hz), δ1 (1–2 Hz), δ2 (2–4 Hz), θ (4.0–8.0 Hz), α (8–16 Hz), β (16–32 Hz), low γ (32–64 Hz), and high γ (64–128 Hz) (Buzsáki and Watson, 2012). The topography of the amplitude-frequency modulation is then plotted using the summation of overall activities at all sensors for respective bands of carriers and AM frequencies (Figure 1H). All HHSA analyses were performed using customized MATLAB (MathWorks) scripts.

### Statistical Analysis

For results visualization, the time dimension of the spectral power was summed to produce a two-dimensional Holo-Hilbert spectrum (AM frequency bins × carrier frequency bins). In this spectrum, the *y*-axis represents AM frequency and the *x*-axis refers to carrier frequency. All trials were then averaged and the data from each group were merged as one dataset. Subsequently, the averaged and merged data of each group was rescaled by the log ratio to the average of all timepoints to elevate the homogeneity to fit a normal distribution for further statistical analysis.

For statistical comparisons, differences of the eye-closed and eye-open condition within groups were examined using paired *t*-test, whereas differences between groups were examined using independent *t*-tests. A two-tailed cluster-based non-parametric permutation test (CBnPP test under *p* < 0.05 with 5,000 permutations) was conducted on the multichannel HHSA spectra (channels × AM frequency bins × carrier frequency bins) for multiple comparisons correction (Maris and Oostenveld, 2007). The neighboring distance between two EEG sensors was defined as 75 mm with 5000 permutations for each test. Though unconventional compared to the Bonferroni or false discovery rate (FDR), it is recognizably efficient for multiple comparison errors (Maris and Oostenveld, 2007). This was done for both NC and PD groups, and further for EPD and LPD groups.

Pearson’s correlation analysis was performed to analyze the linear dependence between two variables. Sex distribution was analyzed using a χ^2^ test. Each set of data was expressed as mean ± standard deviation. All *P*-values were two-tailed, and *P* < 0.05 was considered significant.

### Classification, Feature Extraction and Selection for Machine Learning

Electroencephalography components were extracted from each AM of 26 electrodes, where the ratio between two EEG components was used as a feature. The total number of features was 172,640. Afterward, a correlation analysis was conducted on features, which retained one distinct feature from a cluster of features with a correlation greater than 0.95. Subsets of 100 features from the tens of thousands of remaining features were applied to the LogitBoost algorithm to select the three most prioritized features from every subset. This procedure was iterated until the number of features was reduced to 3, crucial for the function in the final model. In this study, seven common algorithms were employed: LogitBoost, Bagging (Bag), Gentle adaptive boosting (GentleBoost), Decision tree (Tree), support vector machine (SVM), Naïve Bayes and K-Nearest Neighbor, all of which were implemented via the MATLAB software. The models’ performance estimation was further analyzed using receiver operating characteristic (ROC) curves to determine the area under the ROC curve (AUC), and values of sensitivity, specificity, precision, F1 measure, and accuracy.

## Results

### Demographic Features of Parkinson’s Disease Patients

This study recruited 99 patients with PD and 59 cognitively normal subjects as HCs (Table 1). Patients with PD demonstrated significantly higher scores in CDR (PD: 0.38 ± 0.3, HC: 0.22 ± 0.25, *P* = 0.001, Cohen’s *d* = 0.58), BDI-II (PD: 8.14 ± 6.62, HC: 1.68 ± 2.89, *P* < 0.001, Cohen’s *d* = 1.26), HAM-D (PD: 6.17 ± 4.84, HC: 1.63 ± 2.73, *P* < 0.001, Cohen’s *d* = 1.16), PDQ-39 (PD: 31.82 ± 28.16, HC: 6.10 ± 8.24, *P* < 0.001, Cohen’s *d* = 1.24), and NPI (PD: 2.98 ± 4.61, HC: 0.54 ± 1.72, *P* < 0.001, Cohen’s *d* = 0.70), compared with HCs. MMSE (PD: 26.35 ± 4.94, HC: 29.64 ± 9.06, *P* < 0.001, Cohen’s *d* = 0.45) and MoCA (PD: 23.27 ± 6.85, HC: 27.86 ± 2.39, *P* < 0.001, Cohen’s *d* = 0.90) were significantly lower in PD patients compared with HCs. LPD patients were older (EPD: 65.26 ± 10.76 years, LPD: 72.42 ± 9.38 years, *P* = 0.002, Cohen’s *d* = 0.71), had a longer disease duration (EPD: 5.88 ± 8.20 years, LPD: 13.5 ± 5.52 years, *P* < 0.001, Cohen’s *d* = 1.10), greater scores for UPDRS (EPD: 28.92 ± 16.93, LPD: 79.20 ± 39.88, *P* < 0.001, Cohen’s *d* = 2.17) and Hoehn and Yahr stage (EPD: 1.58 ± 0.54, LPD: 2.89 ± 0.46, *P* < 0.001, Cohen’s *d* = 2.61), and greater LEDD (EPD: 467.09 ± 435.39 mg/d, LPD: 1351.63 ± 659.26 mg/d, *P* < 0.001, Cohen’s *d* = 1.58) compared with EPD patients. CDR (EPD: 0.32 ± 0.24, LPD: 0.66 ± 0.37, *P* < 0.001, Cohen’s *d* = 1.09), BDI-II (EPD: 6.23 ± 4.83, LPD: 16.21 ± 7.14, *P* < 0.001, Cohen’s *d* = 1.64), HAM-D (EPD: 5.05 ± 3.62, LPD: 10.89 ± 6.40, *P* < 0.001, Cohen’s *d* = 1.12), PDQ-39 (EPD: 23.20 ± 18.25, LPD: 68.11 ± 33.69, *P* < 0.001, Cohen’s *d* = 1.66), and NPI (EPD: 1.89 ± 2.71, LPD: 7.58 ± 7.47, *P* < 0.001, Cohen’s *d* = 1.01) were significantly greater in patients with LPD compared to those with EPD. Patients with LPD displayed lower MoCA scores (EPD: 24.53 ± 5.73, LPD: 18.0 ± 8.67, *P* < 0.001, Cohen’s *d* = 0.89) and ADL scores (EPD: 99.63 ± 1.55, LPD: 70.0 ± 28.28, *P* < 0.001, Cohen’s *d* = 1.48) compared to those with EPD. Antidepressants were prescribed in three (3.03%) patients with PD and one (1.69%) HC, respectively. Two (2.02%) patients with PD and one (1.69%) HC were treated with antipsychotics.

**TABLE 1:** Clinical characteristics of patients with Parkinson’s disease (PD) in early (EPD) and late (LPD) stages, and healthy controls (HC).

	HC	PD		
		
	(*n* = 59)	EPD (n = 80)	LPD (n = 19)	Total (n = 99)
Sex (female/male)	31/28	39/41	9/10	48/51
Age (years)	66.59 ± 8.03	65.26 ± 10.76	72.42 ± 9.38	66.65 ± 10.85
Duration (years)		5.88 ± 8.20	13.5 ± 5.52	7.37 ± 8.30
Hoehn and Yahr stage		1.58 ± 0.54	2.89 ± 0.46	1.84 ± 0.75
LEDD (mg)		467.09 ± 435.39	1351.63 ± 659.26	642.16 ± 599.59
Antidepressants (%)	1 (1.69)	2 (2.50)	1 (5.79)	3 (3.03)
Antipsychotics (%)	1 (1.69)	0	2 (10.53)	2 (2.02)
UPDRS-total	1.63 ± 2.20	28.92 ± 16.93*	79.20 ± 39.88*#	40.18 ± 28.68*
UPDRS-part III	0.56 ± 1.26	17.51 ± 9.33*	41.79 ± 15.78*#	22.21 ± 14.47*
MMSE	29.64 ± 9.06	27.36 ± 3.85*	22.11 ± 6.66*#	26.35 ± 4.94*
CDR	0.22 ± 0.25	0.32 ± 0.24	0.66 ± 0.37*#	0.38 ± 0.3*
ADL	99.92 ± 0.65	99.63 ± 1.55	70.0 ± 28.28*#	93.94 ± 16.92*
MoCA	27.86 ± 2.39	24.53 ± 5.73*	18.0 ± 8.67*#	23.27 ± 6.85*
BDI-II	1.68 ± 2.89	6.23 ± 4.83*	16.21 ± 7.14*#	8.14 ± 6.62*
HAM-D	1.63 ± 2.73	5.05 ± 3.62*	10.89 ± 6.40*#	6.17 ± 4.84*
PDQ-39	6.10 ± 8.24	23.20 ± 18.25*	68.11 ± 33.69*#	31.82 ± 28.16*
NPI	0.54 ± 1.72	1.89 ± 2.71*	7.58 ± 7.47*#	2.98 ± 4.61*

*Statistically significantly different in comparison with HC.

*#Statistically significantly different in comparison with PD in early stage.*

*ADL, Activities of Daily Living; BDI-II, Beck Depression Inventory II; CDR, Clinical Dementia Rating; HAM-D, Hamilton Depression Rating Scale; LEDD, Levodopa Equivalent Daily Dose; MMSE, Mini-Mental State Examination; MoCA, Montreal Cognitive Assessment; NPI, Neuropsychiatric Inventory Questionnaire; PDQ-39, Parkinson’s Disease Questionnaire; UPDRS, Unified Parkinson’s Disease Rating Scale.*

### Electroencephalography Features of Parkinson’s Disease Patients by Holo-Hilbert Spectral Analysis

Holo-Hilbert spectral analysis showed significant differences in spectral powers between the PD and HC group (Figure 2 and Supplementary Table 1). In the HC group, θ and β bands were dispersed from frontal to occipital regions. Reduced γ bands were observed at frontal and occipital regions (Figure 2A). Although PD group demonstrated spread of energy into θ bands in frontal, central, parietal, and occipital regions similar to HC (Figure 2B), the increased power of θ bands were dispersed to pre- and lateral-frontal regions (Figure 2C). δ2 bands spreading to central, parietal, temporal, and occipital regions were also noted in the PD group. Compared to the HC group, PD patients demonstrated reductions of β bands in frontal and central regions (Figure 2C and Supplementary Table 1). Reduced γ bands, particularly in relatively high amplitude frequencies, were also seen in central, parietal, and temporal regions of PD patients. These results suggest an increase of slowing resting state brain activity into θ and δ2 frequency domains, and reduction of brain activity in β and γ frequency domains, in PD patients.

### Electroencephalography Features Between Early- and Late-Stage Parkinson’s Disease Patients

**FIGURE 3:**
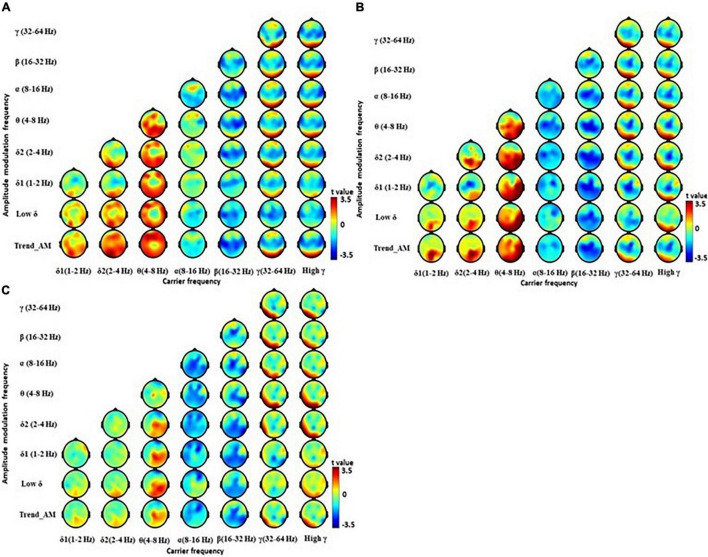
Electroencephalography power difference between PD at early (EPD) and late stage (LPD) using Holo-Hilbert Spectrum Analysis (HHSA). Holo-Hilbert topography (HHT) in the eyes-closed minus the eyes-open condition based on cluster-based non-parametric permutations in the **(A)** EPD minus healthy control (HC), **(B)** LPD minus HC, and **(C)** LPD minus EPD. The color bar denotes *t*-values ranging from blue (–3.5) to red (+3.5).

Holo-Hilbert spectral analysis showed significant differences between the EPD and LPD group (Figure 3 and Supplementary Table 2). Compared with HCs, EPD patients demonstrated dispersed θ and δ bands particularly in relatively low AM frequencies from lateral frontal to occipital regions, and reduced β and γ bands in central and temporal regions (Figure 3A). LPD patients demonstrated increased θ bands from the central frontal to occipital regions, dispersed δ bands in occipital regions, decreased α bands in central and temporal regions, decreased β bands in central, parietal, and occipital regions, and reduced γ bands in the central region (Figure 3B). Compared with EPD group, LPD patients demonstrated reduction of β bands in the posterior central region, and increased θ and δ2 bands in left parietal region (Figure 3C and Supplementary Table 2). These results suggest that LPD patients showed further reduction of fast resting state brain activity in β frequency domains, and an increase of slowing resting state brain activity in θ and δ2 frequency domains, as compared with EPD patients.

### Correlations Between Electroencephalography and Clinical/Neuropsychiatric Features

**FIGURE 4:**
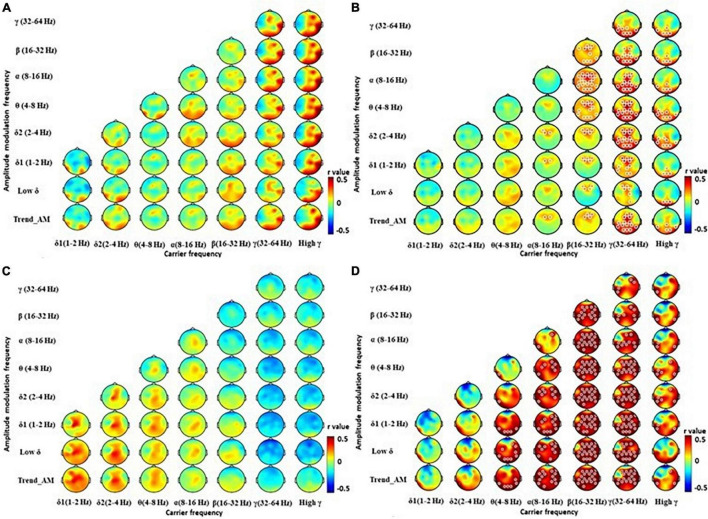
Correlation between powers of Holo-Hilbert Spectrum Analysis (HHSA) and Hamilton Depression Rating Scale (HAM-D). The contrasted HHSA for correlation between HAM-D and **(A)** healthy controls, **(B)** patients with Parkinson’s disease (PD), **(C)** PD patients at early stage (EPD), **(D)** PD patients at late stage (LPD). The white circles indicate that contrast on those EEG channels is statistically significant (*P* < 0.05, cluster permutation test, two-tailed). Color notations depict the *r* value of correlations (shown up to 0.05 for easier visualization purposes).

We further correlated HHSA features with clinical and neuropsychiatric scale scores, where significant results were shown with HAM-D scores (Figure 4). HAM-D scores were significantly positively correlated with β bands in central, parietal, and occipital regions in PD patients, with an *r* value up to and more than 0.7 (Figure 4B). A subgroup analysis showed HAM-D was significantly positively correlated with δ1 and δ2 bands in central regions of EPD patients (Figure 4C). HAM-D and activity from θ to β bands in most of brain regions were significantly positively correlated in LPD patients (Figure 4D). These correlations were not observed in HCs (Figure 4A). Other clinical and neuropsychiatric scales were not correlated with HHSA features of EEG. These results showed fast and slow brain activities, particularly in central, parietal, and occipital regions, could be associated with depressive moods of patients with PD.

### Machine Learning Classification Using Electroencephalography and Neuropsychiatric Features in Parkinson’s Disease Patients

**FIGURE 5:**
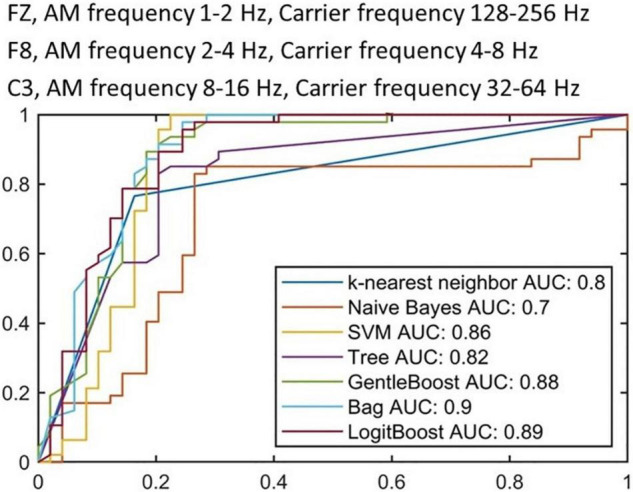
Receiver operating characteristic curves from training stage with 10-fold cross validation. Each ROC curve represents a candidate algorithm (the AUC of all algorithms are higher than 0.7).

We further selected the three most prioritized HHSA features (FZ, AM frequency 1–2 Hz, Carrier frequency 128–256 Hz; F8, AM frequency 2–4 Hz, Carrier frequency 4–8 Hz; C3, AM 8–16 Hz, Carrier frequency 32–64 Hz) that demonstrated significant differences between PD and HC to 7 machine learning algorithms. The sample sizes were 94 for training (PD: 59, HC: 35), with 10-fold cross validation, and 64 for testing (PD: 40, HC: 24). Figure 5 shows the results of applying the training data to each algorithm, with the best result appearing in the “Bag” algorithm with an AUC of 0.90, followed by “LogitBoost” with an AUC of 0.89, and “GentleBoost” with an AUC of 0.88, and AUC of other algorithms were all greater than 0.7. The application of each algorithm to testing data showed that “Bag” demonstrated the highest level of accuracy (0.81), followed by “Tree” (0.80), “LogitBoost” (0.79) and “SVM” (0.74) (Table 2). These results support the potential of implementing machine learning algorithms with HHSA features of EEG as diagnostic tools for PD.

**TABLE 2:** Performance evaluation of classification algorithms deploying PD and HC using features extracted via different analytic methods.

	LogitBoost	Bag	GentleBoost	Tree	SVM	Naïve Bayes	K-Nearest Neighbor
Sensitivity	0.85	0.85	0.90	0.80	0.60	0.20	0.65
Specificity	0.70	0.75	0.60	0.75	0.87	0.95	0.80
Precision	0.70	0.75	0.70	0.80	0.80	0.80	0.79
F1 measure	0.74	0.80	0.77	0.80	0.69	0.32	0.70
Accuracy	0.79	0.81	0.79	0.80	0.74	0.60	0.70

## Discussion

Using the HHSA, we decomposed the EEG signals to produce frequency bands that reflect the natural rhythmic activity of large neural populations. PD patients demonstrated a reduction of β bands in frontal and central regions, and reduction of γ bands in central, parietal, and temporal regions. Compared with EPD patients, LPD patients demonstrated further reduction of β bands in the posterior central region, and increased θ and δ2 bands in left parietal regions. Fast and slow resting state brain activity in the central parietal and occipital regions were positively correlated with HAM-D scores. Machine learning algorithms using three prioritized HHSA features demonstrated good performance when differentiating between PD and HCs, strengthening the application of HHSA in PD diagnosis.

The concept of fuzzy sets (Zadeh, 1965) reliably addresses complexity via the FuzzyEn measure (Chen et al., 2007, 2009) and hence delivers stronger relative reliability and more accurate complexity compared to other entropy-based evaluations (Chen et al., 2009), validating its powerful application to short time series with noise impurity. As EEG signals typically display complex variabilities, indefinite disruption, and great levels of non-linearity and non-stationarity, and other dynamic information (Costa et al., 2005), studying dynamic complexity via entropy better elucidates complex systems (Chen et al., 2007) and potentiates its application clinically (Yang et al., 2013). Patients with Alzheimer’s disease exhibit EEG slowing, reduced complexity of EEG signals, and perturbations in EEG synchrony (Dauwels et al., 2010). These results submit dynamic complexity as a potential bio-signature to monitor health conditions. With high non-linear and non-stationary brainwaves in EEG, especially superimposed trends in signals, the estimation of entropy-based analysis could impact the data by increasing its standard deviation. Thus, to eliminate trend oscillations, the inherent functions (i.e., IMFs) extracted from the EMD are deemed an effective filter for reducing superimposed trends in signals (Huang et al., 1998), as seen in the HHSA. Similarly, the HHSA establishes its advantage in its ability to adapt to EEG signals in time sequences. The HHSA method complements the deficits of traditional spectral analysis and provides a complete informational illustration of non-linear and non-stationary data via the nested EMD and Hilbert–Huang transform (HHT) approach to identify intrinsic amplitude and frequency modulations within non-linear systems (Huang et al., 2016). For non-linear processes, the data contains both amplitude and frequency modulations (intra-mode and inter-mode) engendered via two processes: linear additive or non-linear multiplicative processes. To handle multiplicative processes, extra dimensions in the spectrum are necessary to account for disparities in both the amplitude and frequency modulations concurrently. The HHSA competently accommodates both the additive and multiplicative processes, intra- and inter-mode, stationary and non- stationary, linear and non-linear interactions (Huang et al., 2016). The spectral analysis divulges time-dependent fluctuations and explicitly a measure of the degree of non-linearity within each IMF through the intra-wave frequency variations (Huang, 2014). A core benefit for decomposing the time series into IMFs is that all additive and multiplicative interactions can be separated, extracted and quantified by the first and second layer EMD and HHSA (Huang et al., 1998, 2016; Wu and Huang, 2009). The HHSA can thus methodically define, elucidate and enumerate the linear and non-linear intra- and inter-mode interactions and unfetters spectral analysis from restrictions imposed by Fourier, wavelet or HHT. Since EEG complexity can distinguish patients from health controls, the HHSA is also a promising application for healthcare solutions in the real world.

Overall, the results we obtained concur with previous reports (Tanaka et al., 2000; Kotini et al., 2005; Bosboom et al., 2006; Moazami-Goudarzi et al., 2008) as PD patients exhibited generalized EEG slowing. Recently, Cao et al. (2020) revealed the adaptability of the brain to its environment during visual stimulation using multiscale inherent fuzzy entropy (Cao et al., 2020). The behavioral features of brain electrical activity that decreases in response due to repeated visual stimulation is defined as habituation, and reflects robustness of the brain system (Thompson and Spencer, 1966; Groves and Thompson, 1970). By computing brain complexity in its habituation toward SSVEPs (Cao et al., 2020), they objectively estimated the complexity measure of physiological signals that reveals the robustness of brain systems (Gao et al., 2021) with an essential measure in the crucial features of non-linear neuro-dynamics (Gao et al., 2011). Diseased systems are known to show lower entropy values compared to healthy systems (Takahashi et al., 2010; Cao and Lin, 2018; Cao et al., 2019) and decreased complexity may epitomize reduced brain system integrity, while elevated complexity strongly correlates with stable and accurate behavioral performance (Lippé et al., 2009). Cao et al. (2020) found that EEG complexity increases with increasing visual stimulus times, postulating a strong ability of the brain to tolerate perturbations that ensues in functional or structural systemic modification (Cao et al., 2020). Humans are also able to stop reacting to a stimulus that is no longer biologically relevant (Thompson and Spencer, 1966; Groves and Thompson, 1970), but rather habituate to repeated visual stimulus that no longer have effects. This habituation performance is a form of adaptive behavior, and reflects the robustness of brain systems. In our results using the HHSA, the differences between controls and patients plausibly agree with Cao et al. (2020), as the slowing of EEG in PD patients during the resting state also indicates decreased complexity (Babiloni et al., 2011; Yang et al., 2013). Thus, PD patients may also have decreased habituation since the robustness of their brain systems are compromised.

Parkinson’s disease patients largely exhibited a reduction in higher frequency β and γ bands. This occurred with increment in lower frequency θ and δ bands. In patients with PD, the extensive decline of dopamine leads to abnormal oscillatory activity within the thalamus, further affecting oscillations within the cortex (French and Muthusamy, 2018). The pathophysiologic oscillations of the thalamus (i.e., thalamocortical dysrhythmia) (Jeanmonod et al., 2001), occurs due to thalamic over-inhibition. The lack of dopamine input to the basal ganglion causes the globus pallidus output nucleus to be abnormally active (Levy et al., 2000), and exerts over-inhibition on the ventral lateral and ventral anterior nucleus, through the pallido–thalamic tract (Magnin et al., 2000; Anderson et al., 2003). This overinhibition results in hyperpolarization and deactivation of calcium T-channels in thalamic neurons, and generates low-threshold calcium spike bursts in an inter-burst frequency of ∼4 Hz (Steriade et al., 1990; Jeanmonod et al., 1996; Llinás, 2014). The anatomical and functional coupling between the thalamus and cortex produces high coherence between these structures (Van Horn and Sherman, 2007), yielding overproduction of θ activity in the cortex. Our study consistently showed increased θ bands in the cortex of patients with PD (Figure 2C). The increased brain activity in δ bands, particularly in LPD patients (Soikkeli et al., 1991; Figure 3C), denotes further widespread slowing of activity in PD, which is a marker of bradyphrenia (Brown, 2003; Rowland et al., 2015) as well as cognitive decline and dementia (Bosboom et al., 2006; Stoffers et al., 2007).

The consistent finding of reduced β and γ bands in frontal, central, parietal, and temporal regions in patients with PD (Figure 2C), probably originates from the unilateral sensorimotor cortex (Stancák and Pfurtscheller, 1996; Doyle et al., 2005). This spreads to bilateral sensorimotor regions at movement onset (Neuper et al., 2006), starting from 1000 ms prior to movement onset. This suppression of β bands is likely sustained if the effector is moving (Wheaton et al., 2009). Notably, treating PD patients with levodopa significantly increases β bands, suggesting abnormal β bands (Melgari et al., 2014) as a possible biomarker of motor impairment in patients with PD. A prominent γ band provides a signature of engaged networks. In the sensory cortex, γ bands increase with sensory drive (Henrie and Shapley, 2005), and with a broad range of cognitive phenomena, including perceptual grouping (Tallon-Baudry and Bertrand, 1999) and attention (Fries et al., 2001). The role of reduced γ bands in patients with PD warrants further study.

Correlation analyses with clinical scales revealed significant strong positive correlations only in HAM-D. No other correlation was found between θ bands with overall PD severity (UPDRS, H&Y stage), or cognitive examinations (CDR, MMSE, MoCA). Further studies will be needed to explore the pathophysiological and clinical roles of δ activity in patients with PD. As for the correlation with depression, this is largely reflected by activities in the thalamocortical and cortico-cortical circuits due to altered EEG oscillations (Fingelkurts and Fingelkurts, 2015). In the resting state, depression is associated with increased β bands. Increased β and θ bands are also reported in depressed patients with attentional deficits (Li et al., 2016). Our study identified a positive correlation between β bands and HAM-D scores, clarifying a role of β bands in the severity of depression (Figure 4B). We also found that patients with PD in different stages may demonstrate different correlations between EEG signals and the severity of depression. A positive correlation between δ bands in the central region and HAM-D scores were observed in EPD patients (Figure 4C), while global θ and β bands were positively correlated with HAM-D scores in LPD patients (Figure 4D). These findings indicate different pathophysiological mechanisms of depression present in patients with PD at different stages. Given that LEDD in LPD patients was significantly higher compared to EPD patients, effects of anti-parkinsonian medications on EEG patterns should be considered.

The introduction of machine learning algorithms in EEG analysis provides a potentially easy, accessible, and affordable technique to support the diagnosis of PD. However, the measurement protocols, number of channels, data preprocessing, and feature selection remain inconsistent. Vanneste et al. (2018) applied SVM subsequent to EEG signals processing with standardized low-resolution brain electromagnetic tomography, and found nine featured EEG signals in 31 PD patients and 264 HCs and found an accuracy of 0.94. However, the model performance may have been overestimated due to the imbalance of patients with PD and HCs. Yuvaraj et al. (2018) extracted 13 features in eyes-closed EEG signals in 20 PD patients and 20 HCs by high order spectra. Utilizing the SVM according to these features achieved an accuracy greater than 0.99. However, the relatively small number of subjects raise concerns of overfitting and inadequate generalization. Our method of machine learning considered the fact that EEG signals are irregular and mobile, hence exhibits unpredictability during the classification performance (Abbass et al., 2014). Transfer learning can manage data that violate this hypothesis through manipulating knowledge acquired while learning a given task for solving a different but related task. This obliterates the need to calibrate from the initiating point, yields less noise for transferred information, and depends on prior usable data to proliferate dataset size. By using fuzzy-rule based classification systems, sensible rules can be developed to process EEG activities based on knowledge of neurophysiology and neuroscience, and are therefore explicable. The extraction of intrinsic EEG activities from a neuro-fuzzy model similar to ours considers the fact that EEG signals are non-linear and non-stationary (Jang et al., 2005). A fuzzy inference system (FIS) automatically extracts fuzzy “If-Then” rules from the data and describes which input feature values correspond to which output category (Fabien et al., 2007), permitting the advantage of flexible boundary conditions for BCI applications, EEG pattern classification, and interpreting what the FIS has learned (Sugeno, 1993). Hence, this provides better domain accommodation interpretability and signal processing capability that are particularly advantageous for handling non-linear and non-stationary EEG signals. In PD, Oh et al. (2020) proposed an EEG-based deep learning approach with a convolution neural network (CNN) architecture as a computer-aided diagnostic system and established its possibility in clinical usage for PD detection (Oh et al., 2020). Dunne et al. (2016) presented a specific class of recurrent neural network (RNN) structure termed echo state networks (ESNs) to differentiate EEG signals collected from patients with random eye movement sleep behavioral disorder who ultimately developed PD or Lewy Body Dementia and healthy controls (Dunne et al., 2016). Cao et al. (2020) used inherent fuzzy entropy to study repetitive SSVEPs for analyzing EEG complexity change between migraine phases, while employing the AdaBoost classification with an accuracy of 0.81 ± 0.06 and AUC of 0.87 for differentiating interictal and preictal phase of migraine (Cao et al., 2020). In our study, a relatively large number of patients and HCs were recruited, and limited features were selected to avoid overfitting, adding to the consideration that we incorporated features from the second layer EMD into our method, additional features that can be used for the classification are introduced. The specific electrodes were F8 (AM 2–4 Hz with FM 4–8 Hz), FZ (AM 1–2 Hz with FM 128–256 Hz) and C3 (AM 8–16 Hz with FM 32–64 Hz). The Bag algorithm demonstrated the best accuracy (0.81) compared with other algorithms (Table 2), while the ROC showed an AUC of 0.90 by Bag, followed by 0.79 by GentleBoost and LogitBoost (Figure 5). These findings suggest the potential application of HHSA in preprocessing EEG signals for further diagnosis of PD by machine learning algorithms. Further validation by larger cohorts and refinement of feature extraction methods would be important to improve the performance of these models.

Although our study consolidates the role of HHSA in identification of EEG features in patients with PD, there are some limitations. The numbers of LPD and PDD patients are relatively small. The EEG signals could be affected by use of medications, such as anti-parkinsonian, antidepressants, and anti-psychotics. The single-center nature of our studies lacks external validity. Future multi-center studies with a large number of patients will be required to incorporate our findings into clinical practice.

## Conclusion

Our HHSA method for decomposing and characterizing PD EEG signals permitted the differentiation between matched normal controls and PD patients. Furthermore, the HHSA was sensitive in detecting tendencies toward depression corresponding with a hyperstable regulation of arousal. Features extracted from the HHSA also enabled the distinction of PD from normal controls, specifically in the F8, FZ and C3 electrodes. Further validation will be needed using larger cohorts to refine feature extraction methods to improve the performance of these models, especially to differentiate between the different stages and existence of PD induced dementia.

## Data Availability Statement

Data will be made available from the corresponding author upon reasonable request.

## Ethics Statement

This study involved human participants and was reviewed and approved by the Institutional Review Boards of the Chang Gung Memorial Hospital (ethical license nos: 201801049A3 and 201801051A3). Informed consent was collected from all subjects involved in the study. The patients/participants provided their written informed consent to participate in this study.

## Author Contributions

K-HC, C-MC, and C-HJ: conception, organization, resources, and finalize the manuscript. Y-SL, Y-RW, IF, W-KL, M-LC, NH, and C-HJ: performing EEG and their analysis. K-HC, C-MC, H-CW, and S-NL: recruited patients, examined patients and controls. IF, C-HJ, W-KL, and NH: statistical analysis. K-HC: funding acquisition. K-HC and C-HJ: writing of the first draft. All authors: review and critique. C-MC: supervision.

## Conflict of Interest

The authors declare that the research was conducted in the absence of any commercial or financial relationships that could be construed as a potential conflict of interest.

## Publisher’s Note

All claims expressed in this article are solely those of the authors and do not necessarily represent those of their affiliated organizations, or those of the publisher, the editors and the reviewers. Any product that may be evaluated in this article, or claim that may be made by its manufacturer, is not guaranteed or endorsed by the publisher.
