# Supplementary material for: Evaluating the Different Stages of Parkinson’s Disease Using Electroencephalography With Holo-Hilbert Spectral Analysis
Source: Front Aging Neurosci. 2022 May 10;14:832637. doi: 10.3389/fnagi.2022.832637 (PMC9127298; doi:10.3389/fnagi.2022.832637)
Supplement: Supplementary file 1 [file Table_1.docx]

**Supplementary Tables**

**Supplementary Table 1.** Topographical alterations of amplitude modulation frequencies and the corresponding carrier frequency bands between patients with Parkinson’s disease (PD) and healthy controls (HCs). Topographical registration of amplitude modulation frequencies and the corresponding carrier frequency bands in eye-close minus eye-open conditions, in the two-sample *t*-test based on cluster-based non-parametric permutations for PD-HC. Low δ denotes lower-frequency oscillation. Color notations depict the following for negative power differences: light blue, *P* < 0.01; dark blue, *P* < 0.001, and for positive power differences: yellow, *P* < 0.05; orange, *P* < 0.01, and red, *P* < 0

| **Carrier frequency** | | **δ1 (1-2 Hz)** | | | | | **δ2 (2-4 Hz)** | | | | **θ (4-8 Hz)** | | | | | **α (8-16 Hz)** | | | | | **β (16-32 Hz)** | | | | | | | **γ (32-64 Hz)** | | | | | |
| --- | --- | --- | --- | --- | --- | --- | --- | --- | --- | --- | --- | --- | --- | --- | --- | --- | --- | --- | --- | --- | --- | --- | --- | --- | --- | --- | --- | --- | --- | --- | --- | --- | --- |
| **AM** | | **Low δ** | **δ1** | **Low δ** | **δ1** | **δ2** | | **Low δ** | **δ1** | **δ2** | | **θ** | **Low δ** | **δ1** | **δ2** | | **θ** | **α** | **Low δ** | **δ1** | | **δ2** | **θ** | **α** | **β** | **Low δ** | **δ1** | | **δ2** | **θ** | **α** | **β** | **γ** |
| **Region** | **Electrode** |  | | | | | | | | | | | | | | | | | | | | | | | | | | | | | | | |
| **Frontal** | **FP1** |  |  |  |  |  | |  |  |  | |  |  |  |  | |  |  |  |  | |  |  |  |  |  |  | |  |  |  |  |  |
|  | **FP2** |  |  |  |  |  | |  |  |  | |  |  |  |  | |  |  |  |  | |  |  |  |  |  |  | |  |  |  |  |  |
|  | **F7** |  |  |  |  |  | |  |  |  | |  |  |  |  | |  |  |  |  | |  |  |  |  |  |  | |  |  |  |  |  |
|  | **F3** |  |  |  |  |  | |  |  |  | |  |  |  |  | |  |  |  |  | |  |  |  |  |  |  | |  |  |  |  |  |
|  | **FZ** |  |  |  |  |  | |  |  |  | |  |  |  |  | |  |  |  |  | |  |  |  |  |  |  | |  |  |  |  |  |
|  | **F4** |  |  |  |  |  | |  |  |  | |  |  |  |  | |  |  |  |  | |  |  |  |  |  |  | |  |  |  |  |  |
|  | **F8** |  |  |  |  |  | |  |  |  | |  |  |  |  | |  |  |  |  | |  |  |  |  |  |  | |  |  |  |  |  |
|  | **FC1** |  |  |  |  |  | |  |  |  | |  |  |  |  | |  |  |  |  | |  |  |  |  |  |  | |  |  |  |  |  |
|  | **FC2** |  |  |  |  |  | |  |  |  | |  |  |  |  | |  |  |  |  | |  |  |  |  |  |  | |  |  |  |  |  |
|  | **FC5** |  |  |  |  |  | |  |  |  | |  |  |  |  | |  |  |  |  | |  |  |  |  |  |  | |  |  |  |  |  |
|  | **FC6** |  |  |  |  |  | |  |  |  | |  |  |  |  | |  |  |  |  | |  |  |  |  |  |  | |  |  |  |  |  |
| **Central** | **C3** |  |  |  |  |  | |  |  |  | |  |  |  |  | |  |  |  |  | |  |  |  |  |  |  | |  |  |  |  |  |
|  | **CZ** |  |  |  |  |  | |  |  |  | |  |  |  |  | |  |  |  |  | |  |  |  |  |  |  | |  |  |  |  |  |
|  | **C4** |  |  |  |  |  | |  |  |  | |  |  |  |  | |  |  |  |  | |  |  |  |  |  |  | |  |  |  |  |  |
|  | **CP1** |  |  |  |  |  | |  |  |  | |  |  |  |  | |  |  |  |  | |  |  |  |  |  |  | |  |  |  |  |  |
|  | **CP2** |  |  |  |  |  | |  |  |  | |  |  |  |  | |  |  |  |  | |  |  |  |  |  |  | |  |  |  |  |  |
|  | **CP5** |  |  |  |  |  | |  |  |  | |  |  |  |  | |  |  |  |  | |  |  |  |  |  |  | |  |  |  |  |  |
|  | **CP6** |  |  |  |  |  | |  |  |  | |  |  |  |  | |  |  |  |  | |  |  |  |  |  |  | |  |  |  |  |  |
| **Parietal** | **P3** |  |  |  |  |  | |  |  |  | |  |  |  |  | |  |  |  |  | |  |  |  |  |  |  | |  |  |  |  |  |
|  | **P7** |  |  |  |  |  | |  |  |  | |  |  |  |  | |  |  |  |  | |  |  |  |  |  |  | |  |  |  |  |  |
|  | **PZ** |  |  |  |  |  | |  |  |  | |  |  |  |  | |  |  |  |  | |  |  |  |  |  |  | |  |  |  |  |  |
|  | **P4** |  |  |  |  |  | |  |  |  | |  |  |  |  | |  |  |  |  | |  |  |  |  |  |  | |  |  |  |  |  |
|  | **P8** |  |  |  |  |  | |  |  |  | |  |  |  |  | |  |  |  |  | |  |  |  |  |  |  | |  |  |  |  |  |
| **Occipital** | **O1** |  |  |  |  |  | |  |  |  | |  |  |  |  | |  |  |  |  | |  |  |  |  |  |  | |  |  |  |  |  |
|  | **OZ** |  |  |  |  |  | |  |  |  | |  |  |  |  | |  |  |  |  | |  |  |  |  |  |  | |  |  |  |  |  |
|  | **O2** |  |  |  |  |  | |  |  |  | |  |  |  |  | |  |  |  |  | |  |  |  |  |  |  | |  |  |  |  |  |

**Supplementary Table 2.** Topographical alterations of amplitude modulation frequencies and the corresponding carrier frequency bands between patients with PD at early (EPD) and late stage (LPD). Topographical registration of amplitude modulation frequencies and the corresponding carrier frequency bands in eye-close minus eye-open conditions, in the two-sample *t*-test based on cluster-based non-parametric permutations for LPD-EPD. There is more spread of energy into occipital regions in δ and θ bands in LPD compared to EPD. Low δ denotes lower-frequency oscillation. Color notations depict the following for negative power differences: light blue, *P* < 0.01; dark blue, *P* < 0.001, and for positive power differences: yellow, *P* < 0.05; orange, *P* < 0.01, and red, *P* < 0.00

| Carrier frequency | | δ1 (1-2 Hz) | | δ2 (2-4 Hz) | | | θ (4-8 Hz) | | | | α (8-16 Hz) | | | | | β (16-32 Hz) | | | | | | γ (32-64 Hz) | | | | | | |
| --- | --- | --- | --- | --- | --- | --- | --- | --- | --- | --- | --- | --- | --- | --- | --- | --- | --- | --- | --- | --- | --- | --- | --- | --- | --- | --- | --- | --- |
| AM | | **Low δ** | **δ1** | **Low δ** | **δ1** | **δ2** | **Low δ** | **δ1** | **δ2** | **θ** | **Lo δ** | **δ1** | **δ2** | **θ** | **α** | **Low δ** | **δ1** | **δ2** | **θ** | **α** | **β** | **Low δ** | **δ1** | **δ2** | **θ** | **α** | **β** | **γ** |
| Region | **Electrode** |  | | | | | | | | | | | | | | | | | | | | | | | | | | |
| Frontal | **FP1** |  |  |  |  |  |  |  |  |  |  |  |  |  |  |  |  |  |  |  |  |  |  |  |  |  |  |  |
|  | **FP2** |  |  |  |  |  |  |  |  |  |  |  |  |  |  |  |  |  |  |  |  |  |  |  |  |  |  |  |
|  | **F7** |  |  |  |  |  |  |  |  |  |  |  |  |  |  |  |  |  |  |  |  |  |  |  |  |  |  |  |
|  | **F3** |  |  |  |  |  |  |  |  |  |  |  |  |  |  |  |  |  |  |  |  |  |  |  |  |  |  |  |
|  | **FZ** |  |  |  |  |  |  |  |  |  |  |  |  |  |  |  |  |  |  |  |  |  |  |  |  |  |  |  |
|  | **F4** |  |  |  |  |  |  |  |  |  |  |  |  |  |  |  |  |  |  |  |  |  |  |  |  |  |  |  |
|  | **F8** |  |  |  |  |  |  |  |  |  |  |  |  |  |  |  |  |  |  |  |  |  |  |  |  |  |  |  |
|  | **FC1** |  |  |  |  |  |  |  |  |  |  |  |  |  |  |  |  |  |  |  |  |  |  |  |  |  |  |  |
|  | **FC2** |  |  |  |  |  |  |  |  |  |  |  |  |  |  |  |  |  |  |  |  |  |  |  |  |  |  |  |
|  | **FC5** |  |  |  |  |  |  |  |  |  |  |  |  |  |  |  |  |  |  |  |  |  |  |  |  |  |  |  |
|  | **FC6** |  |  |  |  |  |  |  |  |  |  |  |  |  |  |  |  |  |  |  |  |  |  |  |  |  |  |  |
| Central | **C3** |  |  |  |  |  |  |  |  |  |  |  |  |  |  |  |  |  |  |  |  |  |  |  |  |  |  |  |
|  | **CZ** |  |  |  |  |  |  |  |  |  |  |  |  |  |  |  |  |  |  |  |  |  |  |  |  |  |  |  |
|  | **C4** |  |  |  |  |  |  |  |  |  |  |  |  |  |  |  |  |  |  |  |  |  |  |  |  |  |  |  |
|  | **CP1** |  |  |  |  |  |  |  |  |  |  |  |  |  |  |  |  |  |  |  |  |  |  |  |  |  |  |  |
|  | **CP2** |  |  |  |  |  |  |  |  |  |  |  |  |  |  |  |  |  |  |  |  |  |  |  |  |  |  |  |
|  | **CP5** |  |  |  |  |  |  |  |  |  |  |  |  |  |  |  |  |  |  |  |  |  |  |  |  |  |  |  |
|  | **CP6** |  |  |  |  |  |  |  |  |  |  |  |  |  |  |  |  |  |  |  |  |  |  |  |  |  |  |  |
| Parietal | **P3** |  |  |  |  |  |  |  |  |  |  |  |  |  |  |  |  |  |  |  |  |  |  |  |  |  |  |  |
|  | **P7** |  |  |  |  |  |  |  |  |  |  |  |  |  |  |  |  |  |  |  |  |  |  |  |  |  |  |  |
|  | **PZ** |  |  |  |  |  |  |  |  |  |  |  |  |  |  |  |  |  |  |  |  |  |  |  |  |  |  |  |
|  | **P4** |  |  |  |  |  |  |  |  |  |  |  |  |  |  |  |  |  |  |  |  |  |  |  |  |  |  |  |
|  | **P8** |  |  |  |  |  |  |  |  |  |  |  |  |  |  |  |  |  |  |  |  |  |  |  |  |  |  |  |
| Occipital | **O1** |  |  |  |  |  |  |  |  |  |  |  |  |  |  |  |  |  |  |  |  |  |  |  |  |  |  |  |
|  | **OZ** |  |  |  |  |  |  |  |  |  |  |  |  |  |  |  |  |  |  |  |  |  |  |  |  |  |  |  |
|  | **O2** |  |  |  |  |  |  |  |  |  |  |  |  |  |  |  |  |  |  |  |  |  |  |  |  |  |  |  |
